# Second order motion compensated spin-echo diffusion tensor imaging of the human heart

**DOI:** 10.1186/1532-429X-17-S1-P81

**Published:** 2015-02-03

**Authors:** Christian T Stoeck, Constantin von Deuster, Martin Genet, David Atkinson, Sebastian Kozerke

**Affiliations:** 1 Institute for Biomedical Engineering, University and ETH Zurich, Zurich, Switzerland; 2 Imaging Sciences and Biomedical Engineering, King's College London, London, UK; 3 Centre for Medical Imaging, University College London, London, UK

## Background

Stimulated echo acquisition mode (STEAM) [1] imaging has been used to probe myocardial microstructure in-vivo. However STEAM imaging requires 2 R-R intervals, sophisticated respiratory navigator gating [2] and is subject to myocardial strain [3,4]. Spin-echo (SE) based single-shot diffusion weighted sequences present an appealing alternative [5,6]. In this work the sensitivity to bulk motion of cardiac SE diffusion tensor imaging is addressed by using second order motion compensated (MC) diffusion encoding.

## Methods

**Figure 1:**
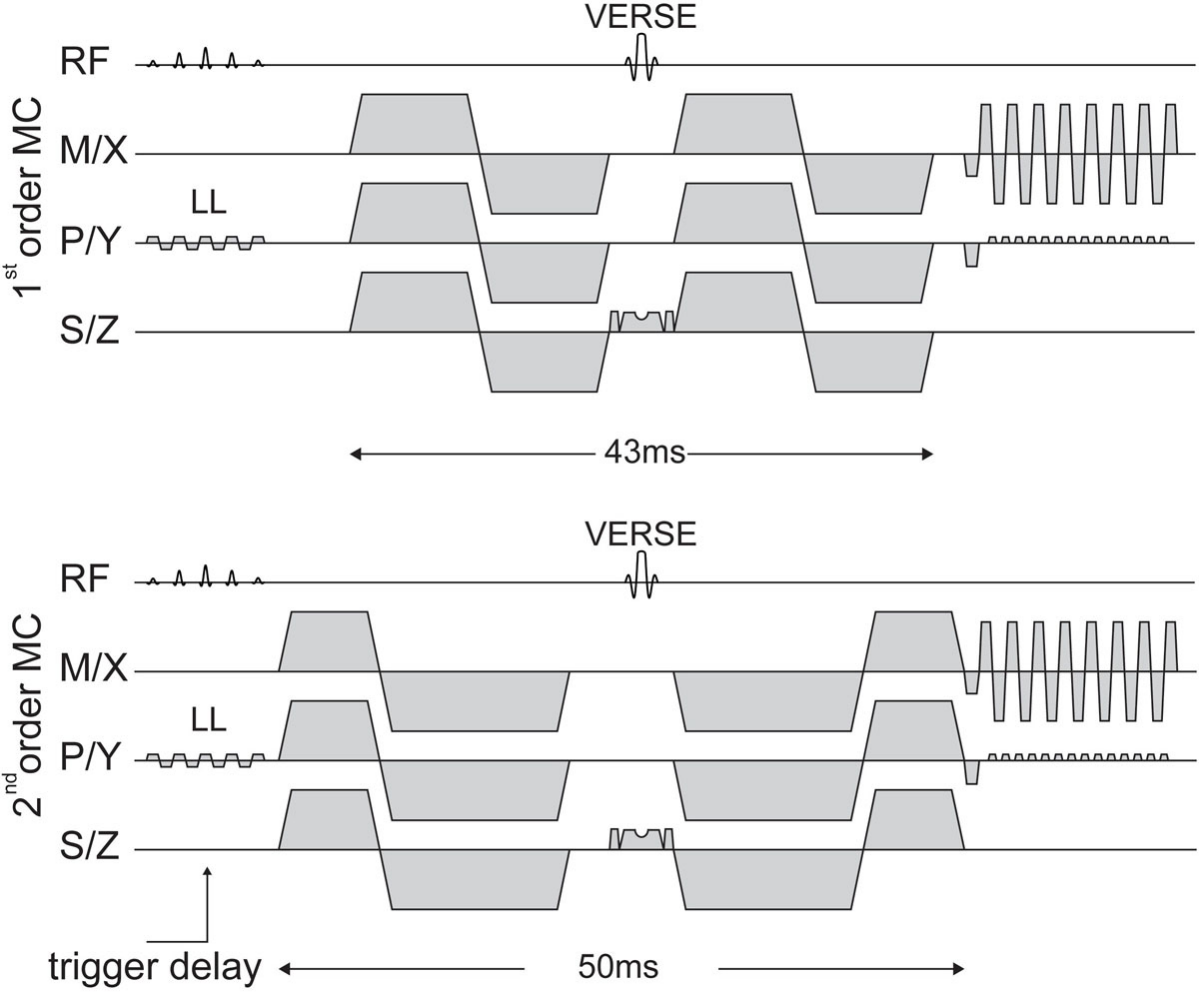
**Sequence diagram of first and second order motion compensated (MC) diffusion imaging.** A spatial spectral reduced field of view (LL) excitation is used for fat suppression. A variable rate selective excitation (VERSE) echo pulse is used for refocussing.

First and second order MC diffusion encoding gradients were incorporated into a cardiac triggered single-shot SE sequence (Figure 1). Imaging was performed on a 1.5T Philips Achieva system (Philips Healthcare, Best, The Netherlands) equipped with gradients delivering 80mT/m@100mT/m/ms. Five healthy volunteers were imaged with navigator-gating during free-breathing with the following parameters: resolution: 2.2×2.2mm^2^, slice thickness: 6mm, local-look FOV: 230×98mm^2^, TR/TE: 1R-R/83ms, two slices (apex/base). Fat suppression was incorporated by spectral-spatial excitation. Three orthogonal diffusion encoding directions (b=450s/mm^2^, 8 averages) were applied at trigger delays ranging from 45ms to peak systole (steps of 10ms). Ten diffusion directions (10 averages, TR: 2R-R) were acquired in an additional session at 38%/47%/56%/66%/75% peak systole. The mean diffusivity (MD) was calculated as function of trigger delay and used as measure for the sensitivity to bulk motion. Helix angles were calculated upon tensor reconstruction.

## Results

**Figure 2:**
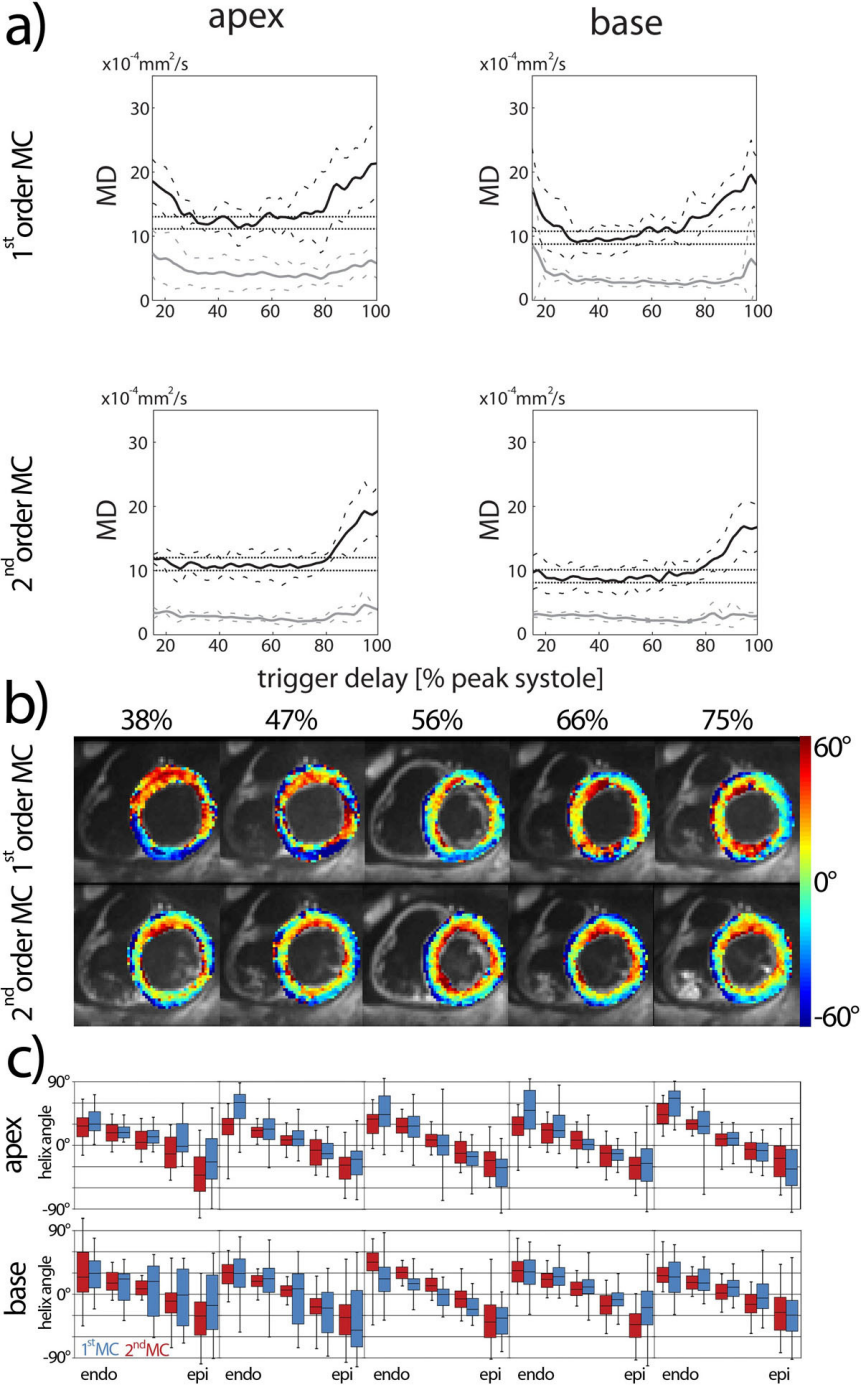
**a) Mean diffusivity (MD) for first and second order motion compensated (MC) diffusion encoding as function of trigger delay.** Black lines represent the mean MD across the myocardium and gray the corresponding standard deviation. Solid lines represent the average across volunteers, dashed lines the corresponding standard deviation. The horizontal dashed lines indicate a range of minimum MD and 2.05×10^-4^mm^2^/s above. b) Example helix angle maps at basal level (top) for different trigger delays (% peak systole) and first as well as second order motion compensated (MC) diffusion encoding. c) Transmural helix angle histograms for apex and base. The box represents the 50% percentile and error bars the 90% percentile across the myocardium at different transmural depths.

MD as function of trigger delay is shown in Figure 2a). Second order MC diffusion encoding yielded an applicable trigger delay range of 15-81% (apical) and 15-77% (basal) of peak systole. For first order MC, the corresponding trigger delay windows were only 30-57% (apical) and 27-56% (basal). Figure 2b) shows a time series of helix angle maps (basal) and the transmural angle histograms (apical/basal) c).

## Conclusions

Second order motion compensated cardiac SE diffusion encoding significantly decreases the sensitivity to bulk motion compared to first order motion compensated diffusion gradients across the heart.

## Funding

Swiss National Science Foundation, grant #CR3213_132671/1, EU FP7 Marie-Curie fellowship to MG, UK EPSRC, grant EP/I018700/1.
